# Fingerprinting of Green Arabica Coffee Volatile Organic Compounds (VOCs): HS-GC-IMS Versus GC × GC-MS

**DOI:** 10.1155/ijfo/1302823

**Published:** 2025-08-22

**Authors:** Matteo Bordiga, Vincenzo Disca, Marcello Manfredi, Elettra Barberis, Francesca Carrà, Luciano Navarini, Valentina Lonzarich, Marco Arlorio

**Affiliations:** ^1^Department of Pharmaceutical Sciences, University of Piemonte Orientale, Novara, Italy; ^2^Department of Translational Medicine, University of Piemonte Orientale, Novara, Italy; ^3^Department of Sciences and Technological Innovation, University of Piemonte Orientale, Alessandria, Italy; ^4^Illycaffè Spa, Trieste, Italy; ^5^Aromalab Illycaffè Spa, Trieste, Italy

**Keywords:** chemometrics, *Coffea arabica*, coffee authentication, GC × GC-MS, HS-GC-IMS, VOCs

## Abstract

This study compared two nontargeted analytical techniques—headspace gas chromatography–ion mobility spectrometry (HS-GC-IMS) and comprehensive two-dimensional gas chromatography–mass spectrometry (GC × GC-MS)—to fingerprint the volatile organic compounds (VOCs) of green *Coffea arabica* beans from Ethiopia, Brazil, Nicaragua, and Guatemala. HS-GC-IMS enabled rapid differentiation of samples, detecting VOC signal regions that effectively clustered samples by origin with minimal preparation. GC × GC-MS offered higher chemical resolution, identifying 98 compounds, including methoxypyrazines, aldehydes, and alcohols, which significantly contributed to interorigin variability. Principal component analysis (PCA) and partial least squares discriminant analysis (PLS-DA) confirmed the capacity of both methods to distinguish geographical origins, with hierarchical clustering highlighting region-specific VOC patterns. HS-GC-IMS proved efficient for high-throughput screening, while GC × GC-MS provided molecular insights into potential aroma precursors. Together, these platforms offer a complementary approach to green coffee authentication and quality control.

## 1. Introduction

Coffee is one of the most consumed beverages globally, with *Coffea arabica* (Arabica) constituting approximately 60% of global coffee production [1]. Arabica is known for its complex flavor profile, characterized by floral and fruity notes, making it highly valued in specialty coffee markets. The chemical makeup of green coffee beans, particularly their volatile organic compounds (VOCs), plays a crucial role in defining the quality and flavor potential of the final roasted product [1]. VOCs in green coffee act as precursors to the compounds that emerge during roasting, including aldehydes, ketones, esters, and pyrazines, which contribute to the coffee's aroma and taste [2]. The composition of these VOCs is strongly influenced by factors such as geographical origin, altitude, and postharvest processing methods [3]. Consequently, VOC profiling has become an essential tool for verifying the geographical origin and quality of coffee beans [4].

The ability to authenticate coffee beans based on their geographical origin is particularly important for high-value single-origin coffees from regions like Ethiopia, Brazil, Nicaragua, and Guatemala, where specific environmental conditions impart unique characteristics to the beans [5].

Marker compounds reported in previous studies include 2-methylpyrazine, 2,3-butanedione, 2-furfurylthiol, methylpropanal, and various alcohols and esters, which have shown region-specific trends. For example, 2-methylpyrazine and 2-furfurylthiol have been linked to coffees from Brazil and Guatemala, while higher levels of linalool and 3-methylbutanol have been associated with Ethiopian. Traditional one-dimensional GC-MS approaches with various sample preconcentration devices have been widely used for coffee VOC profiling. For example, Dong et al. employed headspace solid-phase microextraction coupled with gas chromatography–mass spectrometry (HS-SPME-GC-MS) to analyze green *Coffea robusta* beans from seven Hainan cultivars, identifying 79 volatiles including hydrocarbons, acids, aldehydes, esters, alcohols, and trace levels of pyrazines/pyridine [6]. Similarly, Gancarz et al. used SPME-GC-MS to analyze roasted Arabica from five regions, observing notable levels of nitrogen-containing compounds such as pyridine, alcohols, aldehydes, hydrazides, and acids dominated the aroma profile while associating high pyridine (a bitter off-note) with a particular roasting method, illustrating how VOC analysis can detect roast defects [7].

Recent advancements in nontargeted analytical techniques, such as comprehensive two-dimensional gas chromatography–mass spectrometry (GC × GC-MS) and headspace gas chromatography–ion mobility spectrometry (HS-GC-IMS), have enabled more precise and detailed profiling of coffee VOCs [5]. Unlike traditional targeted methods, which focus on specific compounds, nontargeted approaches allow for the comprehensive characterization of a sample's VOC profile, capturing even subtle differences that may arise due to environmental or processing variations.

GC × GC-MS has emerged as one of the most powerful techniques for VOC analysis due to its superior separation capabilities [8]. This method offers enhanced resolution by separating volatile compounds that coelute in traditional one-dimensional gas chromatography, enabling the detection of trace-level compounds. This has proven particularly useful for identifying VOCs that serve as markers for the geographical origin of coffee. For instance, Lukic et al. [9] used the standard GC-MS for the identification of the basic volatile profile, but the use of GC × GC allowed the identification of a rather larger number of minor and trace volatiles, among which many exhibited a certain discrimination potential.

These unique volatile profiles allow for the authentication of coffee beans and help maintain the integrity of the global coffee trade, where geographic labeling plays a crucial role in determining product value [4, 10].

HS-GC-IMS enables rapid, nondestructive VOC analysis at low temperatures, making it well suited for heat-sensitive compounds in green coffee. Its high throughput allows efficient screening of large sample sets, supporting quality control efforts. Studies such as Chen et al. [11] have shown its effectiveness in differentiating regional coffee samples by detecting variations in alcohols (e.g., 1-hexanol), ketones (e.g., 2-butanone), and sulfur compounds [5]. This rapid method of analysis provides a practical solution for the coffee industry, where quick and accurate assessments of coffee origin and quality are essential for maintaining market value [12, 13].

The nontargeted approaches provided by GC × GC-MS and HS-GC-IMS have resulted in significant advancements in the ability to differentiate coffee from various geographical regions, including Ethiopia, Brazil, Nicaragua, and Guatemala. By identifying specific marker compounds unique to each region, these methods allow for the detailed chemical fingerprinting of coffee beans [14]. Advanced nontargeted VOC profiling by two-dimensional GC and novel detectors has greatly improved the ability to differentiate Arabica coffees by origin. For example, Caporaso et al. achieved ~99% accuracy in classifying single Arabica beans by country (including Ethiopia and Guatemala) using SPME-GC-MS fingerprints [15] and a recent study of Shi et al. showed that HS-GC-IMS (identifying 105 VOCs) could satisfactorily separate coffees from different countries via PLS-DA [16]. These comprehensive VOC fingerprints reveal region-specific markers: Ethiopian coffees tend to be rich in monoterpenes (e.g., linalool and related glycosides and geraniol derivatives) [17], imparting characteristic floral notes, whereas other origins show different profiles. In contrast, roasting-generated volatiles—notably furanic aldehydes such as furfural and 5-methylfurfural—become prominent in intensely roasted beans with sweet, caramel-like aroma notes [18]. Thus, roast degree must be carefully controlled when using VOC profiles to assign origin [15].

This information is crucial for ensuring the authenticity of single-origin coffees, which are becoming increasingly popular among consumers. The ability to trace coffee beans to their geographic origin is also vital for preventing fraud and protecting the reputation of coffee-growing regions known for high-quality production [14, 19].

The primary aim of this study is to perform a comparative evaluation of two nontargeted analytical techniques—HS-GC-IMS and GC × GC-MS—for VOC fingerprinting of green *Coffea arabica* samples from Ethiopia, Brazil, Nicaragua, and Guatemala. The objective is to assess the relative effectiveness, strengths, and limitations of each method in discriminating samples by geographical origin and to identify key volatile markers associated with origin-specific aroma profiles. This comparative approach provides critical insights into the applicability of these techniques for coffee authentication, quality control, and market differentiation in the coffee industry.

## 2. Materials and Methods

### 2.1. Samples

Nine different green wet-processed coffee samples (*C. arabica* L., commercial lots) from batches of the same harvest season and from four different geographical origins (Ethiopia [4], Brazil [3], Nicaragua [1], and Guatemala [1]) were supplied by Illycaffè S.p.A. (Trieste, Italy). The samples with zero primary and secondary defects were selected on the basis of standard internal procedures of sorting and visual appearance, moisture content, screen size, and cup quality. Sample nomenclature is as follows: 80, 84, 170, and 014E from Ethiopia; 014, 046, and 023 from Brazil; 668 from Guatemala; and 1090 from Nicaragua.

### 2.2. HS-GC-IMS Analysis

HS-GC-IMS (FlavourSpec, G.A.S., Dortmund, Germany) was used to assess the volatile composition with an untargeted fingerprinting approach, according to a previous work [3, 20], with minor modifications. Briefly, a 20-mL glass vial was filled with 1.0 g of the sample and then samples were treated for 5 min at 50°C at 500 rpm. Then, in splitless mode, a 300-*μ*L headspace sample was automatically delivered through a 70°C heated syringe. Using an MXT-5 column (15 m × 0.53 mm i.d., 1-*μ*m film thickness; Restek Corporation, Bellefonte, Pennsylvania, United States), the volatile chemicals were separated at 40°C. As the carrier gas, 99.999% pure nitrogen was employed, and the flow rate program was configured as follows: 2 mL/min for 3 min, followed by a 17-min rise to 25 mL/min and a 5-min hold. A 3H ionization source ionized the eluted analytes before driving them to a drift tube, which was operated at a constant temperature of 45°C and voltage of 5 kV. The identification of VOCs was based on the retention index (RI) and drift time (reactive ion peak [RIP] relative) in the GC–IMS library. The analysis software used was VOCal and three plugins (reporter, gallery plot, and dynamic principal component analysis [PCA] plugins). VOCal software was used to view the analytical spectrum and qualitatively and quantitatively analyze the data.

### 2.3. GC × GC-TOF Analysis

A two-dimensional GC coupled to MS was used to characterize volatile molecules using headspace solid-phase microextraction analysis (SPME). A Pegasus BT 4D GC × GC-TOF-MS instrument (LECO Corp., St. Joseph, Michigan, United States) equipped with a LECO dual-stage quad-jet thermal modulator was used. Two grams of the sample was placed into a 20-mL glass vials with septa and equilibrated at 50°C under continuous stirring for 5 min. The volatiles were extracted by SPME, using a 50/35 *μ*m DVB/CAR/PDMS fiber (Supelco Inc., Bellefonte, Pennsylvania, United States). The SPME fiber was preconditioned for 30 min at 270°C and reconditioned between each run to minimize carryover effects.

The first-dimension column was a Stabilwax-DA (Restek Corp., Bellefonte, Pennsylvania) MS capillary column, with an internal diameter of 0.25 mm and a stationary phase film thickness of 0.25 mm, while the second-dimension chromatographic column was a 2 m Rxi-17Sil MS (Restek Corp., Bellefonte, Pennsylvania) with the same diameter and thickness of the first one. High-purity helium (99.9999%) was used as the carrier gas with a flow rate of 1.4 mL/min. The temperature program of the oven was as follows: the initial temperature was set at 50°C for 5 min and then ramped at 4°C/min up to 250°C for 5 min. The secondary column was kept at +15°C above the GC oven temperature of the first column. Electron impact ionization was applied (70 eV). The ion source temperature was set at 250°C; the mass range was 35–550 m/z with an extraction frequency of 32 kHz. The acquisition rates were 200 spectra/s. The modulation periods for both programs were 4 s for the entire run. The LECO Chroma TOF software (Version 5.54) was employed to detect all peaks in the raw two-dimensional chromatogram, applying a signal-to-noise ratio threshold greater than 20. Compound identification was carried out by comparing mass spectra against the NIST MS Search 2.3 library, supplemented with the MoNa Fiehn database. Peaks attributed to stationary phase bleeding were manually eliminated.

### 2.4. Statistical Analysis

PCA and partial least squares discriminant analysis were performed using MetaboAnalyst 5.0 software. Principal components were selected based on their ability to separate sample groups. All the variables (molecules, *n* = 98) and samples (coffee samples, *n* = 9) were included in the dataset. Variables were autoscaled before PCA.

## 3. Results and Discussion

VOCs in green coffee have been widely studied for detecting differentiating elements and the influence of altitude or climatic conditions of different samples. As is well known, these factors directly impact the VOC fingerprint of green Arabica coffee beans as well as the coffee beverage quality [3]. Geographical origin is strictly correlated with different macro and microclimatic conditions, thus representing a critical parameter for coffee quality. The traceability of green coffee can be obtained using different analytical approaches, often completed by appropriate supervised or unsupervised statistical processing of data. The analysis of volatile profile of green coffee is of great interest particularly regarding the evaluation of the fermentation process as reported by Galarza and Figueroa [21], who underline how volatile compounds can vary during fermentation steps [13].

The aim of this work was to establish and compare the usefulness of two hyphenated analytical techniques (GC × GC-TOF and HS-GC-IMS) applied to fingerprint green coffee volatile compounds, evaluating the capacity to cluster specific samples from specific geographical origins.

GC × GC-MS has the ability to explore in depth the complexity of samples, in this case green coffee beans, identifying some key bioactive compounds within the aroma. Considering the results obtained, the application of this analytical method permitted to clearly identify some key odorants of green coffee including methoxypyrazines, aldehydes, alcohols, and hydrocarbons. The supposed presence of 4-(4⁣′-hydroxyphenyl)-2-butanone (raspberry-ketone), which has been previously reported by Akiyama et al. [22] to have a sweet-fruity odor and to be a putative molecular marker of interest in green coffee, was not confirmed in these samples; contrary, it was clearly identified in some raspberry samples, used as positive control.

Moreover, the rapid application of HS-GC-IMS on green beans, an analytical method used in rapid and nondestructive screening [23], was functional in clearly recognizing clusters of samples. HS-GC-IMS provided 2D chromatogram useful to quickly obtain very clear 2D patterns, avoiding any kind of sample preanalytical handlings and processing. Similarly, HS-GC-MS confirmed the absence of raspberry ketone in green coffee samples, reinforcing the GC × GC-MS finding. The comparison of these approaches, processing the overall data (and a selection of the most representative compounds) with PCA, partial least squares discriminant analysis, and hierarchical clustering heatmap, resulted in comparable clustering, clearly permitting to identify the geographical origin of the samples.

**Figure 1:**
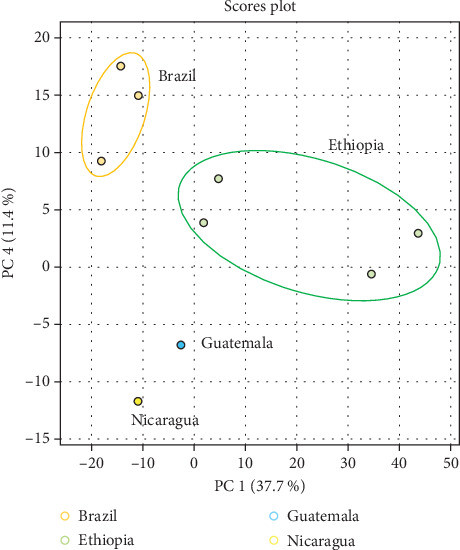
Principal component analysis (PCA) obtained from GC × GC-MS data of coffee samples grouped by geographical origins.

The analysis of green Arabica coffee samples using GC × GC-MS and HS-GC-IMS revealed significant variations in VOCs, both between different areas within the same region and especially between different geographical origins. PCA from GC × GC-MS data (Figure 1) demonstrated that samples are distinctly grouped based on their origin, suggesting that regional environmental factors such as soil composition, altitude, and climate influence the VOC profiles of the coffee beans. Previous studies have well documented the impact of climatic and environmental condition in the composition of aroma precursors in coffee beans [24–26]. This statistical tool was employed to elucidate geographically the distinction between samples and to extract pertinent information from variables that predominantly influence the spatial distribution of the samples.

Samples from Brazil and Ethiopia form clearly defined clusters, indicating significant differences; for instance, coffee beans cultivated at higher altitudes, such as those from Ethiopia, generally exhibit higher concentrations of methoxypyrazines, which contribute to green and grassy flavor notes, while Brazilian green coffee is characterized from a nutty profile with lower acidity [27].

**Figure 2:**
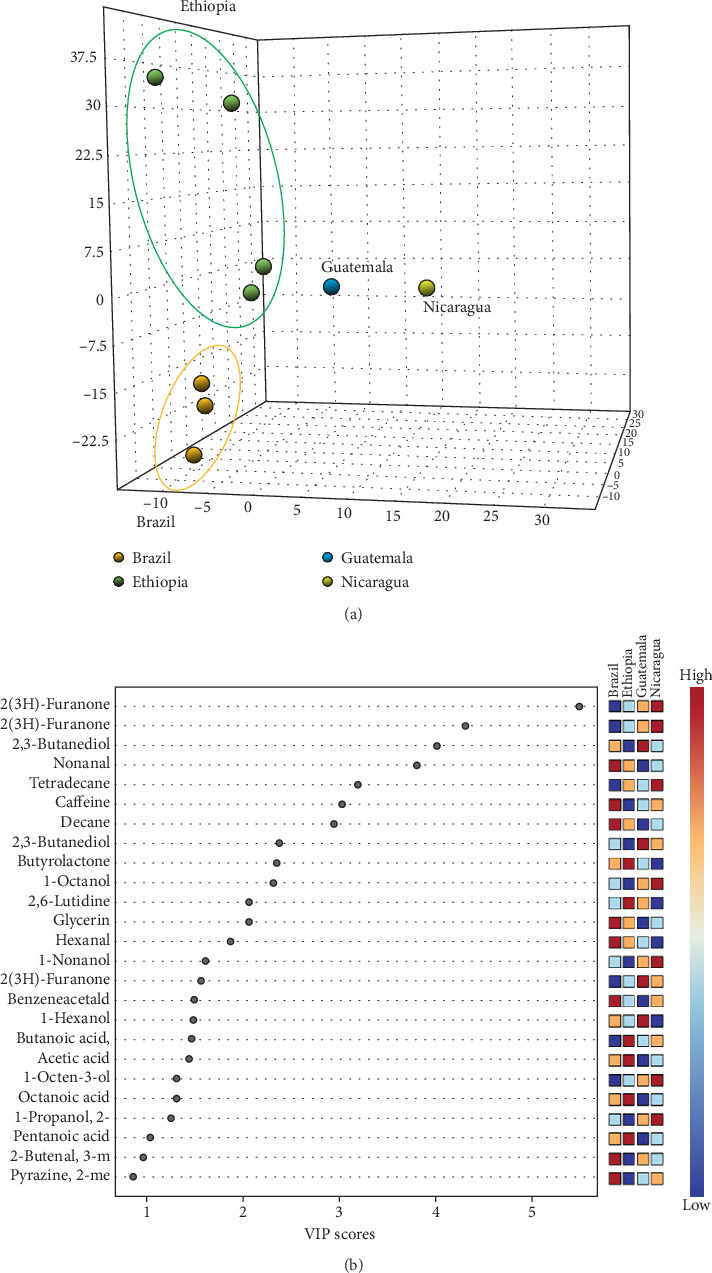
(a) Partial least squares discriminant analysis (PLS-DA) obtained from GC × GC-MS data of coffee samples grouped by geographical origins. (b) Top 25 important features obtained by PLS-DA performed with GC × GC-MS data of coffee samples grouped by geographical origins. The colored boxes on the right indicate the relative importance (red: high; blue: low) of the corresponding metabolite in each coffee group.

Results of PLS-DA further support this conclusion by maximizing the variance between the different groups of coffee samples, thus enhancing the ability to differentiate geographical origins (Figure 2a).

Discriminant analysis methods like PLS-DA, which is a combination of the score and loading plots, effectively illustrating the volatile compounds responsible for specific group differences, are widely employed in food science to identify key variables that contribute to the distinction between sample groups [28]. In this study, the separation obtained with PLS-DA, reported in the variable importance in projection (VIP) measure, highlighted the significant role of specific VOCs, such as 2,3-butanedione, alcohols, and esters, in distinguishing coffee samples (Figure 2b).

Clear clustering is observed, indicating distinct metabolic profiles; Ethiopian samples exhibit strong separation, indicating a distinct metabolic pattern compared to the other regions. In contrast, Guatemalan and Nicaraguan samples are positioned more centrally in the plot; their spatial distribution still indicates discernible variation captured by the model. Overall, the spatial distribution of Central American samples may indicate common agroecological or processing characteristics. This is consistent with the findings of Ortega-Galiván et al. [29], who used similar chemometric methods to distinguish different European hazelnuts based on their origin.

**Figure 3:**
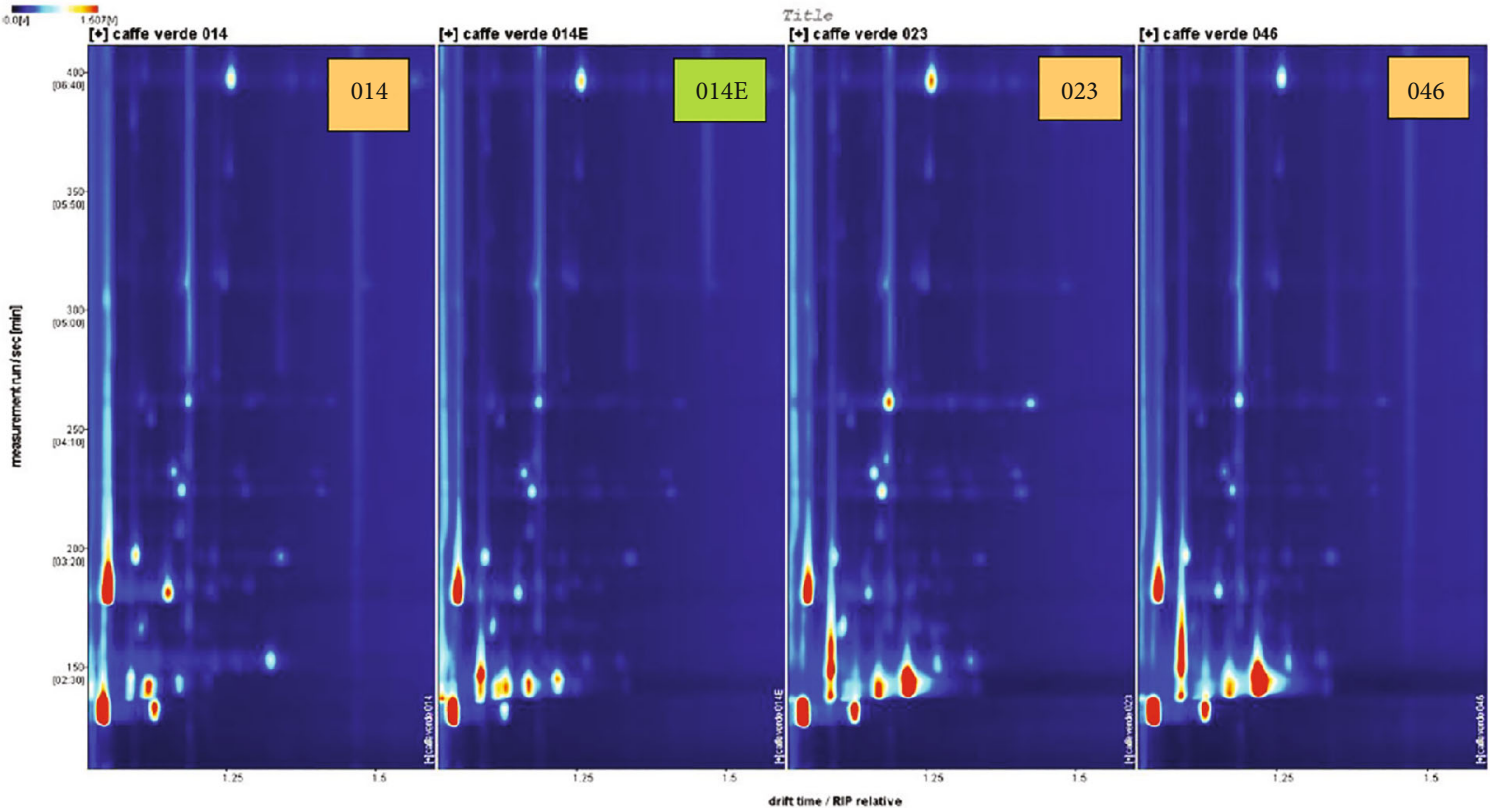
HS-GC-IMS 2D spectrum of 014, 014E, 023, and 046 samples.

**Figure 4:**
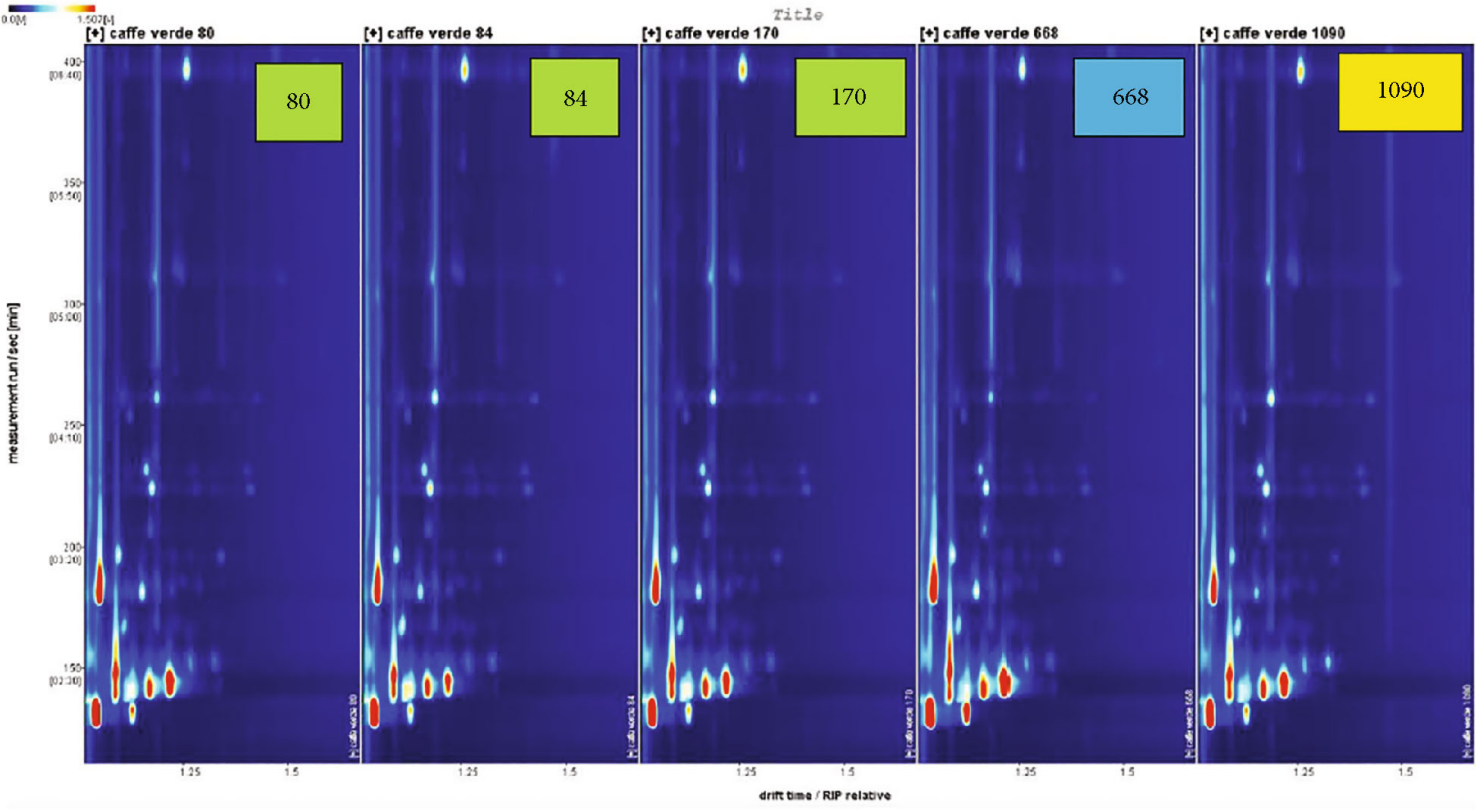
HS-GC-IMS 2D spectrum of 80, 84, 170, 668, and 1090 samples.

Moreover, HS-GC-IMS 2D spectra (Figures 3 and 4) offer rapid and efficient clustering of coffee samples by geographical origin, using minimal sample preparation. Although HS-GC-IMS does not provide the same level of chemical information as GC × GC-MS, it is highly effective for rapid screening of VOCs, making it suitable for industrial applications where quick quality control is essential. The spectra show clustering based on the volatile signatures of each sample, a finding supported by Min et al. [12], who demonstrated the effectiveness of HS-GC-IMS in discriminating food products based on their volatile profiles. The high sensitivity of HS-GC-IMS, particularly for sulfur compounds and terpenes, reinforces its usefulness in distinguishing coffee samples from different origins, as these compounds are known to be influenced by both geographical and postharvest processing factors. Fourteen exhibits distinct peak intensities for certain volatiles and the absence of certain volatile molecules, if compared to the sample cultivated in Ethiopia (014E). Furthermore, there are also differences within samples of the same regions (014, 023, and 046), in terms of intensity of peak. These last findings can depend on different genetic characteristic of the plant, composition of soil, but also microclimate as reported by Cassamo et al. [30], who underline how physical and chemical attributes of green coffee beans can vary depending on genetic characteristic of the plant but also microclimatic condition. Moreover, Mourão et al. [31] show how Arabica coffee beans from various Brazilian areas can have different volatile profile correlated to the altitude, specifically higher altitudes result in a higher aromatic quality of the beans, while lower altitudes provided lower aroma quality. A similar trend is shown in Figure 4, which highlights how samples obtained from different regions can present different volatile markers. The results reinforce the importance of nontargeted VOC fingerprinting in understanding the complexities of coffee flavor and aroma.

**Figure 5:**
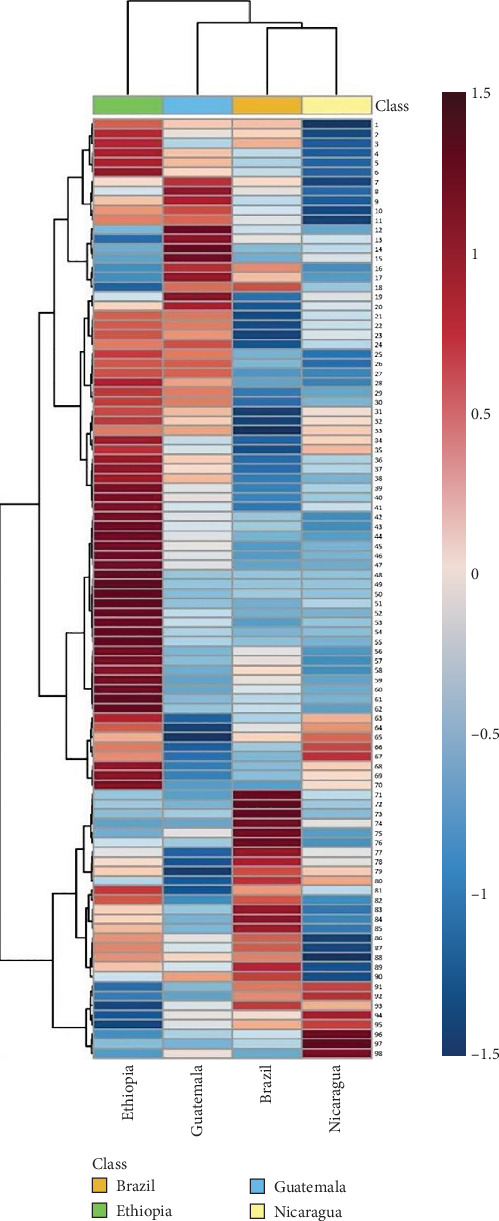
Hierarchical clustering heatmap of the Top 98 identified molecules found in the four geographical varieties of green coffees by GC × GC-MS analysis. Rows represent the 98 volatile compounds analyzed (with compound names reported in Table S1). The color code used for the heatmap visualization is red/blue (for relative maximum and minimum compound abundance of molecule).

The hierarchical clustering heatmap (Figure 5) further supports the PCA and PLS-DA results by illustrating the relationships between the Top 50 identified VOCs and the geographical origins of the coffee samples. This visualization method has been widely used in food chemistry to explore similarities and differences in complex datasets. The clustering patterns observed here are consistent with those reported by Cordero et al. [32], who also identified clear geographical clustering in their analysis of VOCs in coffee. The heatmap confirmed that the chemical composition of green coffee beans is distinct across different regions.

The high altitude slows down the ripening process of the beans, allowing a more intense development of volatile compounds, such as hexanoic acid and furans, which contribute to sweet favor [33]. Moreover, the presence of specific volatile compounds, such as pyrazines, which contributes to roasted, nutty aromas or butanoic acid, that can influence fruity and buttery notes, is tied to the flavor profile of coffee from different regions. Ethiopia's microclimatic variability, combined with the biodiversity of the coffee varieties, favors the presence of complex molecules such as esters and phenolic compounds, which give the coffee an aromatic profile characterized by floral notes and acidity. Brazil, on the other hand, the largest coffee producer in the world, offers different climatic conditions, with lower altitudes than Ethiopia and a tropical climate with well-defined dry and faster growing cycles favors the development of a different, less acid flavor profile. For example, some compounds, such as benzene-1-methoxy (shown in dark red), are highly present in coffee beans from Brazil, while others, like 2-n-heptylfuran, are more prominent in beans from Ethiopia.

**Figure 6:**
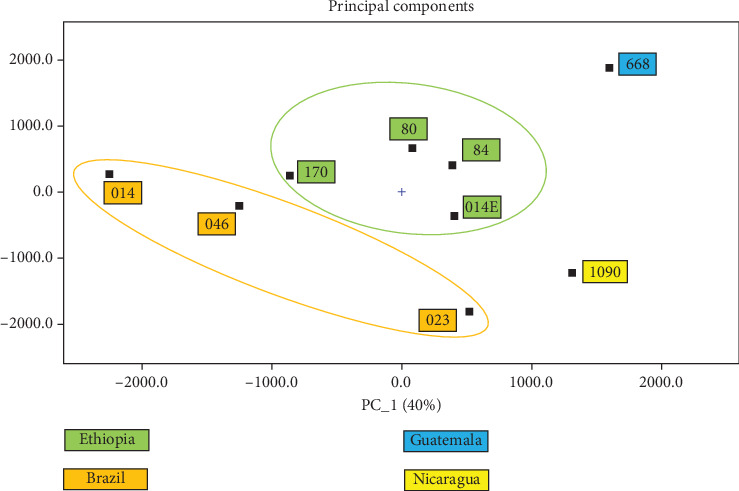
PCA obtained from HS-GC-IMS data of coffee samples grouped by geographical origins.

Finally, PCA based on HS-GC-IMS data (Figure 6) shows comparable results to the GC × GC-MS data, though with less resolution. Despite this, HS-GC-IMS provides a powerful screening tool, especially when rapid analysis is required to discriminate samples, as shown by Valli et al. [34], who demonstrate the potential use of this screening method for olive oil. The PCA plot reveals distinct clustering of the coffee samples by origin, indicating that even without the depth of GC × GC-MS, HS-GC-IMS can still effectively capture the key volatile differences between samples. This underscores the complementary nature of these two techniques, with GC × GC-MS offering detailed molecular insights and HS-GC-IMS providing faster, high-throughput analysis suitable for industrial settings.

The combined application of GC × GC-MS and HS-GC-IMS in this study highlights the importance of a multiplatform approach in VOC analysis. GC × GC-MS provides comprehensive chemical profiling, enabling the identification of specific bioactive compounds like methoxypyrazines, while HS-GC-IMS offers a practical, rapid screening tool with adequate sensitivity to distinguish between geographical origins.

In the analysis of VOCs from *Coffea arabica* grown in Ethiopia, Brazil, Nicaragua, and Guatemala, key differences in VOC profiles can serve as effective markers for geographical discrimination. Studies employing using advanced analytical techniques such as HS-SPME-GC-MS and gas chromatography–ion mobility spectrometry (GC-IMS) provide compelling evidence for the unique metabolomic signatures characteristic of coffee beans from different regions.

One study focusing on Ethiopian coffee highlighted that geographical factors such as altitude, rainfall, and temperature significantly influence the volatile profiles of coffee beans. This research, conducted by Urugo et al. [35], identified a range of 23 volatiles, with aldehydes (39%), terpenes (26%), and alcohols (17.3%) being the most prominent. The composition of these compounds exhibits significant variation according to the altitude and agroecological conditions of the coffee-growing regions, enabling the clear differentiation of coffee samples from diverse Ethiopian regions. The VOC profile, heavily weighted by aldehydes and terpenes, underscores the distinct aromatic characteristics influenced by local environmental conditions [35].

Similarly, research conducted by Zakidou et al. [36] on coffee from Brazil and Peru emphasized the role of pyrazine and furan derivatives, which are key products of the Maillard reaction during roasting. Brazilian coffee exhibited high levels of pyrazines (up to 39.1%), conferring strong nutty and cocoa-like aromas, while Peruvian coffee samples showed greater concentrations of aldehydes and esters, which contributed to their floral and citrus sensory profiles. The chemometric analysis, including PCA, allowed for the effective separation of coffee samples based on these key volatiles, thus demonstrating the significant influence of geographical origin on VOC composition [36].

In the broader context of coffee storage and the potential degradation of VOCs over time, Min et al. [12] used GC-IMS to examine how storage conditions impact the volatile composition of green coffee beans. They identified 38 VOCs, including alcohols, aldehydes, esters, and ketones. Acrolein and butanoic acid were particularly useful in distinguishing the storage times of different samples, while stable compounds such as butanal and dimethyl sulfide remained consistent, indicating their potential role as markers for identifying coffee variety or origin. The use of PCA further confirmed that VOC profiles could effectively discriminate beans stored under varying conditions, although limitations were noted regarding the impact of storage on VOC stability [12].

Additionally, metabolomics as a tool for the authentication of coffee has gained traction, as demonstrated by De León-Solis et al. [4]. This study explored the VOC profiles of coffee from multiple regions using GC-MS and highlighted the potential of volatile metabolite profiling to distinguish coffee beans based on geographical origin. In particular, the study found that Ethiopian coffee beans had distinct profiles rich in aldehydes like hexanal, which were particularly abundant in beans grown at higher altitudes. These results underscore the strong correlation between environmental factors and metabolomic composition, enhancing the traceability and authenticity of specialty coffees [4].

Instrumentally, both HS-SPME-GC-MS and GC-IMS have proven to be effective tools for volatile compound analysis in coffee. HS-SPME-GC-MS allows for a robust profiling of both volatile and semivolatile compounds, while GC-IMS is particularly advantageous for analyzing VOCs at lower temperatures, thus preserving the sample's natural volatile state. However, each technique presents certain limitations. For instance, while HS-SPME-GC-MS is widely recognized for its precision and ability to identify a large number of compounds, it can be labor-intensive and requires careful sample preparation to avoid potential artifacts. Furthermore, GC-IMS, despite being less demanding in terms of sample pretreatment, has limitations in terms of compound resolution when compared to mass spectrometry, especially when dealing with complex mixtures like coffee.

The application of these techniques has been instrumental in unraveling the complex web of volatile interactions that define the aroma of coffee. For example, De Vivo et al. [37] demonstrated the power of static headspace gas chromatography–mass spectrometry (SHS-GC/MS) in distinguishing Arabica coffees from Brazil and Peru based on their aldehyde and ester content. Brazilian Arabica was found to be rich in aldehydes such as hexanal, while Peruvian coffee had a more complex ester profile, contributing to its floral and fruity notes. The chemometric models employed, particularly linear discriminant analysis (LDA), were successful in classifying coffee based on these volatile markers with a high degree of accuracy, underscoring the utility of VOC profiling in coffee authentication [37].

Despite the robust nature of these analytical tools, there are inherent challenges in interpreting VOC data, particularly when trying to account for variables such as roasting levels, bean storage conditions, and environmental factors. For instance, as noted in the work by Zakidou et al. [36], while roasting profiles can enhance the differentiation of VOCs based on origin, they can also obscure some regional markers due to the intense thermal degradation of compounds during darker roasting processes. This highlights the need for a carefully tailored approach to both roasting and analysis in order to preserve the key volatile signatures linked to geographical origin.

Overall, the discrimination of *Coffea arabica* from Ethiopia, Brazil, Nicaragua, and Guatemala through VOC analysis shows promising results. Studies consistently demonstrate that specific volatile compounds, such as pyrazines, aldehydes, esters, and terpenes, play a crucial role in defining the geographical and sensory identity of coffee. Instruments like HS-SPME-GC-MS and GC-IMS are essential for this analysis, though careful consideration of their limitations is necessary to optimize results. These findings not only support the use of VOC profiling in the assessment of coffee authenticity and quality but also highlight the potential for advanced chemometric techniques, such as PCA and LDA, in improving regional classification.

## 4. Conclusions

These approaches emphasize the usefulness of the hyphenated multiplatform approach as analytical tool, preliminary to data mining. Both GC × GC-TOF and HS-GC-IMS analysis allowed identifying specific clusters of samples of known geographical origin, also permitting some considerations regarding aroma precursors. Even if GC × GC-TOF permits to obtain a more in-depth and detailed molecular profiling by identifying key odorants, HS-GC-IMS, particularly, reduced the time of analysis by allowing the direct rapid analysis of green beans in vials, opening up new perspectives for quality control of green coffee.

## Data Availability

The original datasets generated from the study are available from the corresponding author on reasonable request.
