# Supplementary material for: Fingerprinting of Green Arabica Coffee Volatile Organic Compounds (VOCs): HS-GC-IMS Versus GC × GC-MS
Source: Int J Food Sci. 2025 Aug 22;2025:1302823. doi: 10.1155/ijfo/1302823 (PMC12396915; doi:10.1155/ijfo/1302823)
Supplement: Supporting Information — Additional supporting information can be found online in the Supporting Information section. Table S1: Volatile organic compounds and relative code. [file 1302823.f1.docx]

Supporting Information

**Fingerprinting of Green Arabica Coffee Volatile Organic Compounds (VOCs): HS-GC-IMS versus GCxGC-MS**

Matteo Bordiga*^1^, Vincenzo Disca^1^, Marcello Manfredi^2^, Elettra Barberis^3^, Francesca Carrà^1^, Luciano Navarini^4^, Valentina Lonzarich^4^ and Marco Arlorio^1^

**Table S1.** Volatile Organic Compounds and relative code.

| **Compound** | **Code** |
| --- | --- |
| 2-Methoxy-3-isopropylpyrazine | 1 |
| 6,10,14-Trimethylpentadecan-2-one | 2 |
| Butan-1-ol | 3 |
| 3,4-Dimethoxybenzaldehyde | 4 |
| 3-Methyldihydrofuran-2(3H)-one | 5 |
| 3-Phenylfuran | 6 |
| Furan-2-ylmethanol | 7 |
| Hexan-1-ol | 8 |
| Cyclohex-2-en-1-one | 9 |
| Dihydrofuran-2(3H)-one | 10 |
| 5-Methyldihydrofuran-2(3H)-one | 11 |
| Butane-2,3-diol | 12 |
| Octan-1-ol | 13 |
| 3-Ethyl-2,5-dimethylpyrazine | 14 |
| 2-Methylpropan-1-ol | 15 |
| Pyrazine | 16 |
| 1,3,7-Trimethylpurine-2,6-dione | 17 |
| (2R,3R)-Butane-2,3-diol | 18 |
| 2-Phenylethanol | 19 |
| Hexanoic acid | 20 |
| Nonanoic acid | 21 |
| Decanoic acid | 22 |
| 4-Vinyl-2-methoxyphenol | 23 |
| 3-Hydroxy-2-methyl-4H-pyran-4-one | 24 |
| 4-Hydroxy-3-methoxybenzaldehyde | 25 |
| Ethyl hexadecanoate | 26 |
| Methyl hexadecanoate | 27 |
| 5-Pentyldihydrofuran-2(3H)-one | 28 |
| 2-Pentylfuran | 29 |
| 5-Methyldihydrofuran-2(3H)-one | 30 |
| Indole | 31 |
| (3E,5E)-Octa-3,5-dien-2-one | 32 |
| 1-(1H-Pyrrol-2-yl)ethanone | 33 |
| (E)-Non-2-enal | 34 |
| Nonan-2-one | 35 |
| 3-Methylbutanoic acid | 36 |
| 2,3-Dihydrobenzofuran | 37 |
| 5-Pentyldihydrofuran-2(3H)-one | 38 |
| 5-Methylfuran-2-carbaldehyde | 39 |
| 3-Methylbut-2-enoic acid | 40 |
| 3-Methylpentanoic acid | 41 |
| Pentadecanal | 42 |
| Oct-1-en-3-ol | 43 |
| 1-(5-Methylfuran-2-yl)ethanone | 44 |
| Methyl 2-aminobenzoate | 45 |
| Dodecanoic acid | 46 |
| 1-(1-Methyl-1H-pyrrol-2-yl)ethanone | 47 |
| Hexadecanoic acid | 48 |
| 1-Methyl-4-(1-methylethenyl)cyclohexene | 49 |
| 5H-Furan-2-one | 50 |
| Pentanoic acid | 51 |
| Benzoic acid | 52 |
| (2E,4E)-Nonadienal | 53 |
| Cyclopent-2-en-1-one | 54 |
| Dodecan-1-ol | 55 |
| Dodecanal | 56 |
| 2,6-Dimethylpyridine | 57 |
| (R)-4,4,7a-Trimethyl-5,6,7,7a-tetrahydro-2H-benzofuran-2-one | 58 |
| Phenol | 59 |
| 1-(5-Methylfuran-2-yl)butan-2-one | 60 |
| (E)-Oct-2-enal | 61 |
| Dodecan-2-one | 62 |
| (E)-Oct-2-enoic acid | 63 |
| (2E,4E)-Nonadienal | 64 |
| Heptanoic acid | 65 |
| Benzaldehyde | 66 |
| Acetic acid | 67 |
| Octanoic acid | 68 |
| 3,7-Dimethylocta-1,6-dien-3-ol | 69 |
| (R)-p-Menth-1-en-8-ol | 70 |
| Methyl 2-hydroxybenzoate | 71 |
| Nonan-1-ol | 72 |
| 3-Methylbut-2-enal | 73 |
| 6-Methylhept-5-en-2-one | 74 |
| Propane-1,2,3-triol | 75 |
| Pyrrole-2-carbaldehyde | 76 |
| 3-Methylphenol | 77 |
| 3,4-Dimethylfuran-2,5-dione | 78 |
| Heptanal | 79 |
| (E)-4-(2,6,6-Trimethylcyclohex-1-en-1-yl)but-3-en-2-one | 80 |
| Furan-2-carbaldehyde | 81 |
| Octan-3-ol | 82 |
| Decanal | 83 |
| 2-Methylprop-2-enoic acid | 84 |
| Butanoic acid | 85 |
| Benzothiazole | 86 |
| 3-Ethyl-4-methylpyrrole-2,5-dione | 87 |
| Hexanal | 88 |
| Methylpyrazine | 89 |
| Heptan-2-ol | 90 |
| 2-Methoxy-3-methylpyrazine | 91 |
| Phenylacetaldehyde | 92 |
| Nonanal | 93 |
| 2-Methoxy-3-(sec-butyl)pyrazine | 94 |
| 2-Methoxy-3-(isobutyl)pyrazine | 95 |
| Octanal | 96 |
| 1,2,4,5-Tetramethylbenzene | 97 |
| 2-Ethylhexanoic acid | 98 |
